# A possible blood plasma biomarker for early-stage Alzheimer’s disease

**DOI:** 10.1371/journal.pone.0267407

**Published:** 2022-04-21

**Authors:** Sandra Anne Banack, Aleksandra C. Stark, Paul Alan Cox

**Affiliations:** 1 Brain Chemistry Labs, Jackson, Wyoming, United States of America; 2 Dartmouth-Hitchock Medical Center, Lebanon, New Hampshire, United States of America; Alanya Alaaddin Keykubat University: Alanya Alaaddin Keykubat Universitesi, TURKEY

## Abstract

We sought to identify a usable biomarker from blood samples to characterize early-stage Alzheimer’s disease (AD) patients, in order to facilitate rapid diagnosis, early therapeutic intervention, and monitoring of clinical trials. We compared metabolites from blood plasma in early-stage Alzheimer’s disease patients with blood plasma from healthy controls using two different analytical platforms: Amino Acid Analyzer and Tandem Mass-Spectrometer. Early-stage Alzheimer’s patient blood samples were obtained during an FDA-approved Phase IIa clinical trial (Clinicaltrial.gov NCT03062449). Participants included 25 early-stage Alzheimer’s patients and 25 healthy controls in the United States. We measured concentrations of 2-aminoethyl dihydrogen phosphate and taurine in blood plasma samples. We found that plasma concentrations of a phospholipid metabolite, 2-aminoethyl dihydrogen phosphate, normalized by taurine concentrations, distinguish blood samples of patients with early-stage AD. This possible new Alzheimer’s biomarker may supplement clinical diagnosis for early detection of the disease.

## Introduction

Onset of Alzheimer’s disease (AD) symptoms is correlated with accumulations of misfolded proteins and protein fragments, particularly amyloidβ42 (Aβ42) plaques and a dense tauopathy of neurofibrillary tangles (NFTs) composed of hyperphosphorylated tau deposits in specific brain regions [1]. There is a latency period between the initiation of AD-type neuropathology in the brain and onset of clinical symptoms [2]. Discovery of new ways of diagnosing AD in the earliest stages, particularly those present during the latency period [3], could lead to new types of treatment, including reconsideration of previously failed drugs [4].

Various biomarkers associated with later stages of AD have been suggested [5] including cerebrospinal fluid (CSF) and plasma biomarkers indicative of amyloid deposition, neuronal damage and loss, and the formation of NFTs, notably phosphorylated-tau (P-tau), Aβ42, total-tau (T-tau), as well as neurofilament light protein (NFL) [6, 7], while plasma concentrations of Aβ40 and Aβ42 may not be as useful in diagnosing AD [6, 8]. Biomarkers based on imaging assessing amyloid-beta plaques (PiB-PET scans), tau deposits (tau-PET), brain atrophy (structural MRI), memory-related activity patterns (fMRI), and decreased glucose metabolism (FDG-PET) have also been proposed [5, 9]. Nucleic acid biomarkers for AD [10, 11], similar to those for ALS [12], have also been proposed [13–15].

We have sponsored an FDA-approved Phase IIa clinical trial of L-serine at the Department of Neurology, Geisel School of Medicine, Dartmouth (Clinicaltrials.gov identifier number NCT03062449) for early-stage Alzheimer’s disease patients. At the time they receive their initial diagnosis, based on the Clinical Dementia Rating (CDR) score, patients are offered entry into the clinical trial. We hypothesized that a unique metabolic biomarker of early Alzheimer’s disease could be identified by examining the physiological amino acids and nitrogen containing compounds within these early disease state blood samples. Using an automated Amino Acid Analyzer along with confirmation from tandem mass-spectroscopy, we examined metabolites displaying clear differences between AD and control blood plasma samples. We found that the concentration of 2-aminoethyl dihydrogen phosphate normalized by taurine concentrations in blood plasma samples reliably identifies early-stage AD patients.

## Materials and methods

We compared initial blood plasma samples collected from the antecubital area of the arm from early-stage Alzheimer’s disease patients (n = 25; 4 females, 21 males; Clinical Dementia Rating Scale 0.5 +/- 0.23) with blood plasma from healthy controls (n = 25; 5 females, 20 males), with analysis blinded. Plasma came from initial blood draws from our Phase IIa human clinical trial for early-stage Alzheimer’s patients, and control samples (Innovative Research Inc., Novi, Michigan, USA). The control subjects had a mean age of 39 (range 20–62 years) while the Alzheimer’s patients had a mean age of 71 years (range 57–82). The study was approved by the IRB (Dartmouth Hitchcock Medical School, NCT03062449, Innovative Research Inc, FDA Approval, #3003372368). Informed consent was obtained from all participants. All methods were performed in accordance with the relevant guidelines and regulations.

Blood plasma was collected in K2-EDTA tubes and centrifuged immediately at 2000 x g for 15 minutes at 4°C. Time between blood collection and freezing was less than 1 hour and the sample was stored at -80°C and shipped on dry ice. The plasma sample was thawed at 4°C and combined with an equal volume of cold 10% (w/v) trichloroacetic acid (TCA, CAS 76-03-9, respectively, Sigma-Aldrich, St. Louis, MO). The sample was left to precipitate at room temperature for two hours followed by centrifugation at 14,000 x g for 5 min. The supernatant was removed by pipette and filtered using a centrifuge filter (0.22 μm PVDF, Millipore Ultrafree-MC-GV, Darmstadt, Germany) at 14,000 x g for 5 min.

The underivatized sample (20 μL) was injected into a Hitachi Amino Acid Analyzer L8900 equipped with a Hitachi Reaction column (PN 855–3533, Hitachi High-Tech America, Inc. Dallas, TX) at 135°C, a high-speed physiological fluid analysis analytical column Li-form resin #2622SC 6 mm ID x 40 L 060928C (PN855-4515), and AmmoniaFilter column (Ion exchange 4.6 x 40 Column #2650L, PN 855–3523). Hitachi pre-made buffers (Hitachi High-Tech America, Inc. Dallas, TX) were used as follows: (B1) PF-1/AN0-5031, (B2) PF-2/AN0-5032, (B3) PF-3/AN0-5033, (B4) PF-4/AN0-5034, (B6) PF-5/AN0-5035, (R1) ninhydrin solution (Wako Chemicals #29970501, Fujifilm Wako Pure Chemical Corporation, Osaka, Japan), and (R2) ninhydrin-buffer of lithium acetate dihydrate (Wako Chemicals 29970501). Wash solutions included (B5) 5% ethanol (95%, Fisher Scientific #22-032-106, Hampton, NH), (R3) 10% methanol (≥ 99.9% (Chromasolv; 34885-4x4, Honeywell Burdick & Jackson, Muskegon, MI), and (C1) 10% methanol. Separation was achieved with a flow rate for pump 1 of 0.54 mL/min and 0.47 mL/min for pump 2, reactor column set to 135°C, and a 146 min gradient elution: 0.00 min = 100% B1, column oven temp 35°C, 50% R1, 50% R2; 16.0 min = 100% B1; 16.1 min = 81% B1, 19% B2, column oven 58°C; 41.0 min = column oven 32°C; 57.0 min = column oven 70°C; 69.5 min = column oven 65°C; 70.0 min = 15% B1, 75% B2, 10% B3; 88.0 min = column oven 60°C; 94.0 min = 20% B2, 80% B4; 94.1 min = 25% B2, 75% B4; 109.0 min = 25% B2, 75% B4, column oven 70°C; 109.1 min = 100% B4; 123.0 min = 100% B4; 123.1 min = 100% B6; 127.0 min = 50% R1, 50% R2; 127.1 min = 100% R3; 129.0 min = 100% B6, 129.1 min = 100% B1; 131.0 min = column oven 35°C; 132.0 min = 100% R3; 132.1 min = 50% R1, 50% R2; 146.0 min = 100% B1. Amino acid standards (Sigma A6407 –acidic amino acids and neutral amino acids + Sigma A6282– basic amino acids, St. Louis, MO) were mixed to equal concentrations and complete standard curves run at concentrations of 5, 10, 25, 50 100, 250, 500, 1000 μmol/L. All amino acids curves were linear within this range (R^2^>/ = 99%). In addition, retention time checks were conducted for 2-aminoethyl dihydrogen phosphate (Sigma-Aldrich P0503-1G, St. Louis, MO) and taurine (Sigma-Aldrich T0625-10G). A lower end curve (1.4, 3.5, 7.1, 14.2, 70.9 μmol/L) demonstrated that 2-aminoethyl dihydrogen phosphate was linear to 1.4 μmol/L (R^2^ = 99.9%). The limits of detection (LOD) and lower limits of quantification (LOQ) were calculated using the EPA method [16]. LOD for 2-aminoethyl dihydrogen phosphate and taurine were 0.9 and 5.3 μmol/L respectively for this method. The LOQ was 2.5 and 10.2 μmol/L for 2-aminoethyl dihydrogen phosphate and taurine respectively for this method.

Confirmation of 2-aminoethyl dihydrogen phosphate in the samples was conducted through tandem mass spectrometry. Plasma samples from AD patients and controls, with analysis blinded as to treatment, were deproteinated as above and derivatized with 6-aminoquinolyl-N-hydroxysuccinimidyl carbamate (AQC, WAT052880, Waters Corp, Milford, MA) following manufacturers recommendations. AD blood samples were combined as were control samples and analyzed on a Thermo TSQ Quantiva triple quadrupole mass spectrometer with multiple injections per sample. The mass spectrometer was equipped with a Thermo Vanquish pump, autosampler, H-ESI probe, and heated column compartment set to 65°C. An OPTON-10005 Genius 3022 dual N2 generator (Peak Scientific, Billerica, MA) supplied purified nitrogen to the mass spectrometer. Separation was achieved using a Kinetex C-18 column (1.7 μm 100 A, 100 x 2.1 mm, 00D-4475-AN, Phenomenex, Torrance, CA); and gradient elution (flow rate 0.5 ml/min, mobile phase A = 20 mM ammonium acetate (Fisher Scientific A11450, Hampton, NH) ≥ 99% adjusted to pH 5.0 with glacial acetic acid (Fisher Scientific A385-500), B = 100% methanol ≥ 99.9% (Chromasolv; 34885-4x4, Honeywell Burdick & Jackson, Muskegon, MI): initial conditions 99.8% A, 1.0 min 99.8% A, 1.5 min 40% A, 2.0 min 25%A, 2.5 min 25%A, 2.6 min 10% A, 5.5 min 10%A, 6 min 98%A, 8 min 98%A all set with curve 5. The mass spectrometer was run in positive mode (4400 V) with the following settings: spray voltage = static; sheath gas = 5.6 Arb; aux gas = 23.5 Arb; sweep gas = 1.2 Arb; ion transfer tube = 150°C; vaporization temperature = 450°C; Q1 resolution = 0.7 FWHM; Q3 resolution = 0.7 FWHM; and CID gas = 2 mTorr. Scans for derivatized 2-aminoethyl dihydrogen phosphate were conducted with the following transitions (312 to 171.2 *m/z* collision energy (CE) 17.1, 312 to 142 *m/z* CE 17.1, 312 to 312 *m/z* CE 2, RF lens of 90). This experiment was repeated a second time to ensure reproducibility. The limit of detection (LOD) for this method was 0.004 μg/ml and the lower limit of quantification (LOQ) was 0.01 μg/ml. A standard curve was prepared with concentrations 20 pg/ml, 40 pg/ml, 0.2 ng/ml, 0.4 ng/ml, 2 ng/ml, 4 ng/ml, 10 ng/ml, 20 ng/ml, 0.1 μg/ml, 0.2 μg/ml, and linearity had an R^2^ value of 99%.

**Fig 1:**
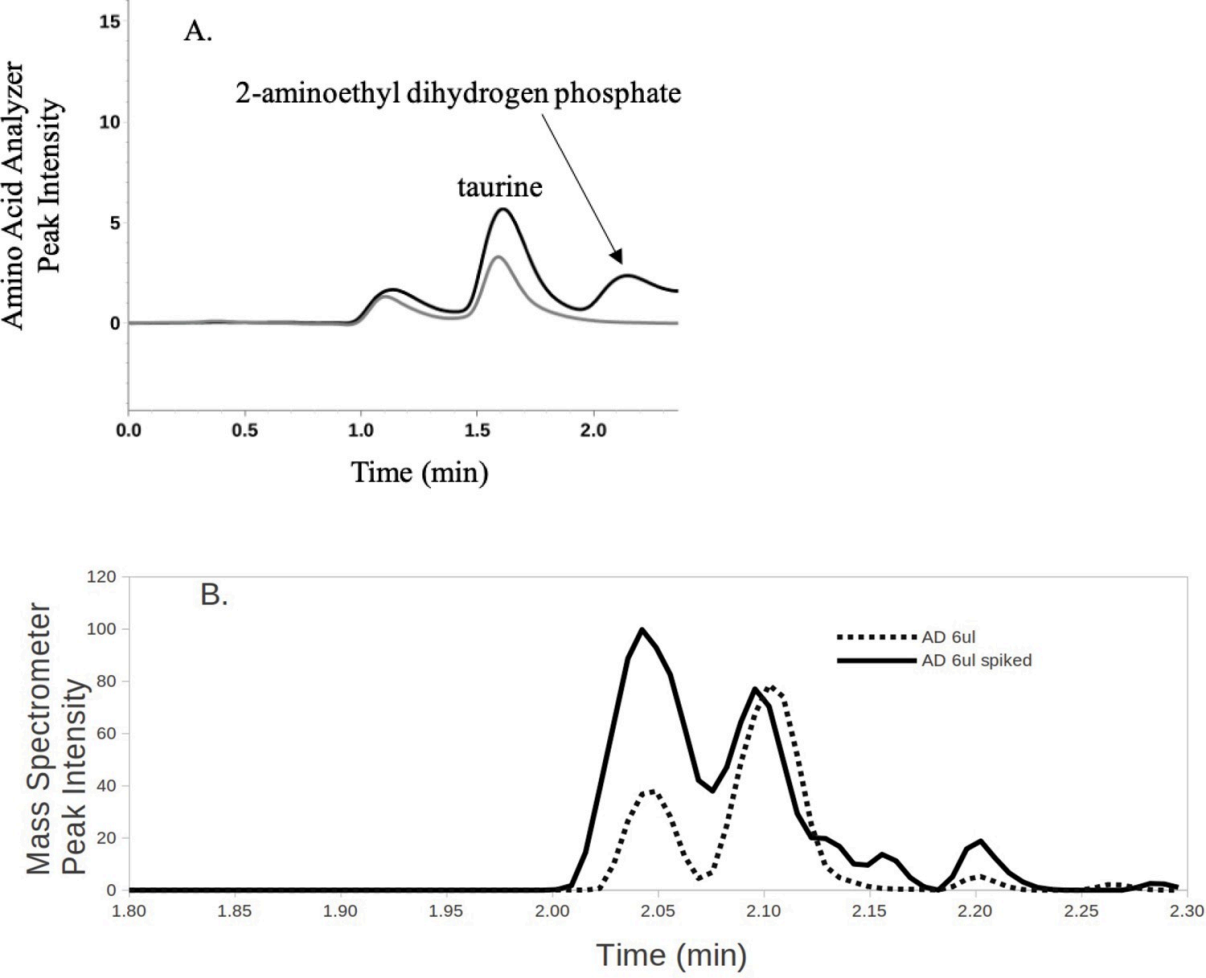
Representative chromatogram from the hitachi Amino Acid Analyzer method. **A.** Taurine elutes at 1.6 min. 2-aminoethyl dihydrogen phosphate elutes at 2.1 min. Dark line is a representative Alzheimer’s patient sample. Grey line shows the absence of 2-aminoethyl dihydrogen phosphate in a control sample which is considered not detectable. Quantification was achieved by measuring the area under the curve in relation to known standard reference concentrations. **B.** Representative chromatogram from the tandem mass spectrometer method with 2-aminoethyl dihydrogen phosphate eluting at 2.05 min. The dotted line is an Alzheimer’s patient sample with an injection volume of 6 μl. The overlaid solid line represents the same sample spiked with a 2-aminoethyl dihydrogen phosphate standard. The identity of the peak at 2.1 min is unknown, however, this peak does not change as a result of the addition of 2-aminoethyl dihydrogen phosphate standard which confirms the identity of the peak at 2.05 min.

The concentration of 2-aminoethyl dihydrogen phosphate, which elutes at 2.1 minutes on the Amino Acid Analyzer chromatogram, was normalized for each sample by the concentration of taurine, an aminosulfonic acid which elutes immediately before at 1.6 minutes (Fig 1A). A ratio of 2-aminoethyl dihydrogen phosphate to taurine was used because of the close proximity of the two chromatographic peaks. When taurine was large in a patient sample (which could possibly arise from hemolysis of the blood sample or the ingestion of taurine rich beverages), it lifted the chromatographic baseline and artificially raised the adjacent 2-aminoethyl dihydrogen phosphate chromatographic peak area. In order to rectify this problem we used the ratio of 2-aminoethyl dihydrogen phosphate to taurine as the final measurement. Therefore, the ratio of 2-aminoethyl dihydrogen phosphate to taurine was calculated for each of the control and Alzheimer’s patient samples. When 2-aminoethyl dihydrogen phosphate was not detected in the sample, the ratio was assigned a 0.0 value. Scatter plots indicated the data were not normally distributed, so the ratios of 2-aminoethyl dihydrogen phosphate to taurine were transformed using sin^−1^(*x*), where *x* is the original ratio. Two hypotheses were tested using a two-tailed *t*-test for samples with unequal variances:

H_0_: The normalized means of 2-aminoethyl dihydrogen phosphate are equal for early-stage Alzheimer’s disease patients and controls.

H_1_: The normalized means of 2-aminoethyl dihydrogen phosphate are different for early-stage Alzheimer’s disease patients and controls.

## Results

**Fig 2:**
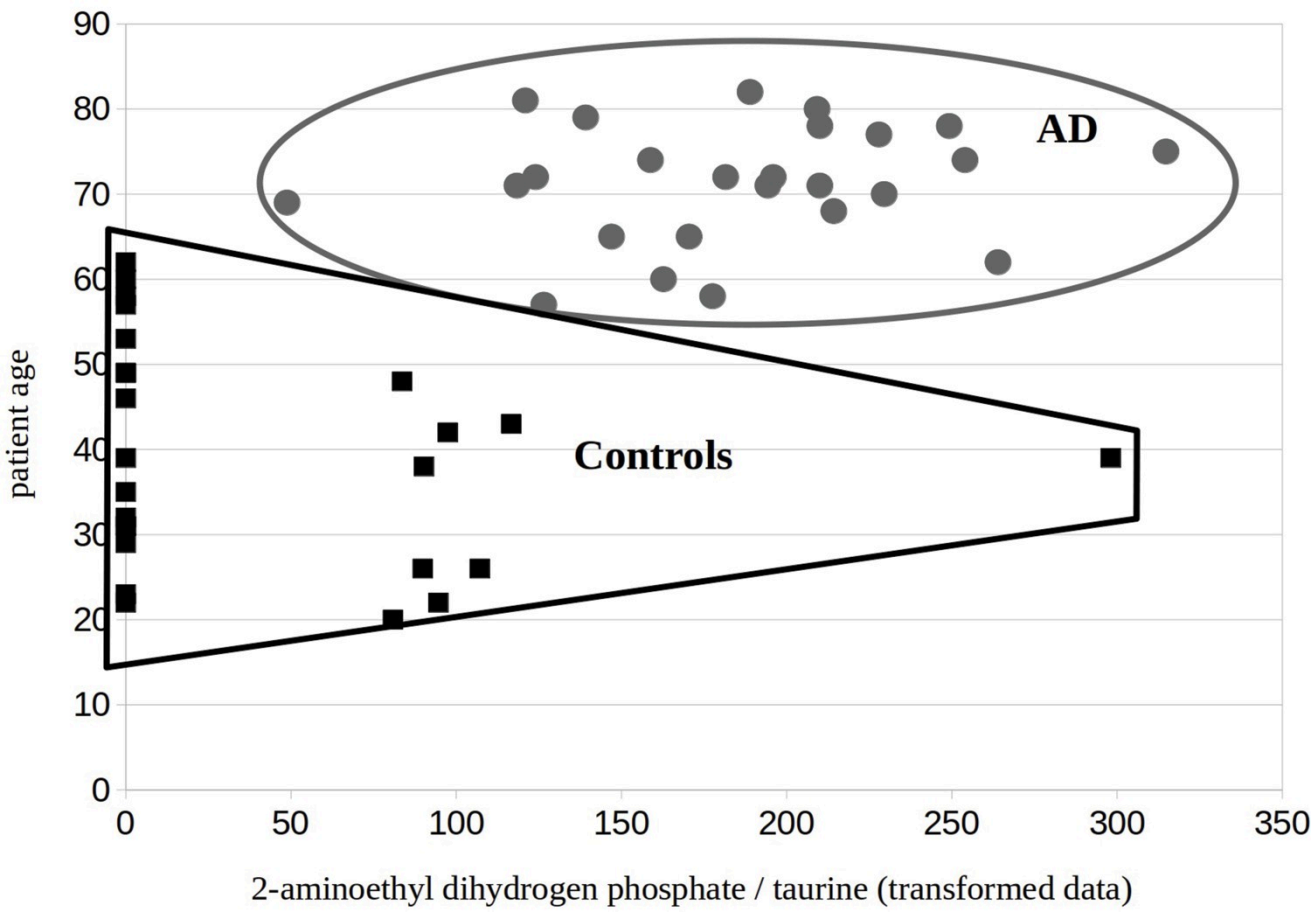
Scatter plot of patient age versus arcsin ratio of 2-aminoethyl dihydrogen phosphate / taurine). Squares are healthy controls (n = 25) and circles are Alzheimer’s patients (n = 25). The arcsin ratio does not correlate with age, however, the incidence of Alzheimer’s disease does increase with patient age.

The mean ratio of 2-aminoethyl dihydrogen phosphate to taurine for Alzheimer’s patients was 0.37 (sd = 0.13) compared to a mean of 0.07 for controls (sd = 0.12) (Table 1). The data were transformed using sin^−1^(*x*) because the data were not normally distributed. The mean of the sin^−1^(*x*) transformed ratio of 2-aminoethyl dihydrogen phosphate to taurine for controls was 42.3 (sd = 69.9), while the mean of the sin^−1^(*x*) transformed ratios for early-stage Alzheimer’s patients was 185.5 (sd = 57.0) with a *t* statistic of -7.9, df = 48. H_0_ was therefore rejected, with p<0.000000001. We note that the absence of an analyte detection does not mean that it is not present, only that while using the stated methods that it is below the limit of detection. Since a value below the LOD approximates a zero compared to the high concentrations reported in other samples (See Fig 1A for a visual representation of the data), we used a zero for computational purposes in these cases (Table 1). Therefore, the concentration of 2-aminoethyl dihydrogen phosphate normalized by taurine in blood draws significantly distinguishes early-stage Alzheimer’s patients from controls (Figs 1A and 2).

**Table 1:** Concentration (μmoles/L) of 2-aminoethyl dihydrogen phosphate and taurine in blood plasma of controls and Alzheimer’s patients calculated using the Amino Acid Analyzer method. The lower limit of detection (LOD) for 2-aminoethyl dihydrogen phosphate using this method was 0.9 μmol/L. The lower limit of quantification (LOQ) for taurine was 10.2 μmol/L. This represents a detectable peak but one below the ability of the method to accurately quantify. When 2-aminoethyl dihydrogen phosphate was not-detectable (ND), ratios were assigned a 0.0 value.

Treatment	2-aminoethyl dihydrogen phosphate	Taurine	Ratio	Treatment	2-aminoethyl dihydrogen phosphate	Taurine	Ratio
Control	ND	60.6	0.0	AD	18.6	56.7	0.3
Control	ND	26.0	0.0	AD	20.7	48.5	0.4
Control	ND	16.6	0.0	AD	20.4	38.5	0.5
Control	ND	19.5	0.0	AD	21.2	86.9	0.2
Control	ND	<10.2	0.0	AD	22.9	58.4	0.4
Control	ND	<10.2	0.0	AD	20.0	55.2	0.4
Control	ND	<10.2	0.0	AD	22.6	52.1	0.4
Control	ND	<10.2	0.0	AD	17.8	49.0	0.4
Control	ND	12.7	0.0	AD	18.7	63.0	0.3
Control	ND	27.7	0.0	AD	21.6	68.6	0.3
Control	ND	<10.2	0.0	AD	23.5	76.0	0.3
Control	35.7	174.3	0.2	AD	22.6	81.5	0.3
Control	36.4	72.4	0.5	AD	23.7	64.0	0.4
Control	52.5	356.2	0.1	AD	46.3	61.3	0.8
Control	52.5	332.0	0.2	AD	9.4	45.3	0.2
Control	37.4	218.2	0.2	AD	9.7	26.6	0.4
Control	ND	44.1	0.0	AD	12.3	31.3	0.4
Control	57.2	303.7	0.2	AD	10.6	31.5	0.3
Control	ND	78.9	0.0	AD	10.3	30.4	0.3
Control	46.7	280.3	0.2	AD	8.4	39.6	0.2
Control	ND	62.1	0.0	AD	9.2	32.3	0.3
Control	49.5	311.4	0.2	AD	7.8	35.2	0.2
Control	ND	43.6	0.0	AD	12.2	47.6	0.3
Control	57.4	402.7	0.1	AD	9.2	20.4	0.5
Control	ND	36.2	0.0	AD	8.0	12.1	0.7

Confirmation of the presence of 2-aminoethyl dihydrogen phosphate in the blood plasma of early-stage AD patients was conducted by tandem mass spectrometry (Fig 1B). 2-aminoethyl dihydrogen phosphate typically was present in 5–10 times higher concentrations in the samples from early-stage AD patients than in samples from controls. This orthogonal method confirms the analytical results from the Amino Acid Analyzer.

## Discussion

2-aminoethyl dihydrogen phosphate is the IUPAC name for a molecule previously referred to in the literature by other names including *O*-phosphorylethanolamine, colamine phosphoric acid, ethanolamine O-phosphate 2, *O*-phosphoethanolamine, *O*-phosphocolamine, and colamine phosphoric acid. This molecule is important in the structure and function of cellular membranes. It is a precursor in the biosynthesis of phosphatidylethanolamine and phosphatidylcholine. It is essential for the formation of mammalian glycosylphosphatidylinositol-anchored proteins (GPI) which bind other proteins to the plasma membrane [17]. GPI may play a role in cellular communication, cellular signaling, signal transduction, and lipid raft transports [18]. The proximate source of the 2-aminoethyl dihydrogen phosphate in GPI synthesis is phosphatidylethanolamine [19].

In cell culture, 2-aminoethyl dihydrogen phosphate has been shown to inhibit mitochondrial respiration and induce apoptosis by disrupting the mitochondrial membrane potential [20]. Physiological effects of increased 2-aminoethyl dihydrogen phosphate concentrations in the blood are not known. However, brain concentrations were reported to be lower in AD patients than in controls [21]. In that study, 2-aminoethyl dihydrogen phosphate concentrations were significantly lower in the temporal cortex (64%, Brodmann area 21), frontal cortex (48%, Brodmann area 9), and hippocampus (40%) but not in the parietal (Brodmann area 3–12) or occipital cortices (Brodmann area 17) of AD patients. In brain tissue and CSF from Huntington’s disease patients, 2-aminoethyl dihydrogen phosphate was lower in concentration in comparison with control tissues. Significant differences were found in the caudate, putamen, and nucleus accumbens but not the frontal cortex of Huntington’s patients [22]. Similarly, a significant decrease in 2-aminoethyl dihydrogen phosphate has also been noted in the putamen of Parkinson’s disease (PD) patients in comparison to control brain tissue based on proton magnetic resonance spectroscopy imagining studies [23]. Hattingen et al. [23] suggest that this reflects reduced membrane turnover as a result of impaired mitochondrial function, which may be a contributing factor in AD [24]. The importance of 2-aminoethyl dihydrogen phosphate as a substrate in the synthesis of cellular membranes and its increased concentration in the blood, but not in the brain tissues of AD patients, suggests that further study of this molecule might lead to new insights into progressive neurodegeneration.

Normal concentrations of 2-aminoethyl dihydrogen phosphate for healthy adults range from “not detected” to up to 69 μmoles/L [25–28]. The Mayo clinic suggests normal 2-aminoethyl dihydrogen phosphate concentrations to be <12 μmoles/L for adults 18 and older, <5 μmoles/L for children ages 2–17 years, and <6 μmoles/L for children under the age of two (https://neurology.testcatalog.org/show/AAQP). Our study suggests that the concentration of 2-aminoethyl dihydrogen phosphate normalized by taurine concentrations in blood plasma samples could potentially be added to the CDR scale as a diagnostic tool for early-stage AD.

Elevated 2-aminoethyl dihydrogen phosphate levels, primarily in urine, also occur in patients diagnosed with a rare inherited disease known as hypophosphatasia [29]. The disease is thought to be caused by mutations in the tissue-nonspecific alkaline phosphatase (TNSALP) gene [29].

If the sphingolipid biosynthesis pathway is disrupted in early-stage AD, an increase in the downstream 2-aminoethyl dihydrogen phosphate could result. A need for additional L-serine to drive the pathway forward could also occur, based on the *de novo* sphingolipid biosynthesis pathway where serine palmitoyltransferase catalyzes the reaction of L-serine with palmitoyl-Coenzyme A to form sphingoid bases. Breakdown of both sphinganine and sphingosine leads to the production of 2-aminoethyl dihydrogen phosphate [30]. *In vitro*, an increase in sphinganine kinase decreases free sphinganine which results in the disruption of axonal growth in cultured hippocampal neurons [31]. Sphingolipid metabolism is thought to be tightly regulated as the metabolites play a role in cellular signal transduction [31].

There are limitations to our study. Since there is evidence of Alzheimer’s latency prior to diagnosis [2] measurable in years, with increasing probability of undiagnosed Alzheimer’s with age, the control subjects had lower mean age than the Alzheimer’s patients. It is possible that patients categorized as early-stage Alzheimer’s in this study suffered from a different form of dementia since neuropathological brain studies were not available to confirm diagnosis. To determine if elevated 2-aminoethyl dihydrogen phosphate is a consequence of normal aging, a longitudinal study in a larger elderly cohort could test whether 2-aminoethanol dihydrogen phosphate concentrations normalized by taurine concentrations accurately predicts which patients convert into Alzheimer’s. We are currently planning such a larger study of Mild Cognitive Impairment patients [IND 155785] which could facilitate such a comparison. Furthermore, it would be informative to analyze 2-aminoethyl dihydrogen phosphate in blood plasma samples of healthy controls and map this analyte relative to age in addition to testing blood plasma samples of patients with other progressive neurodegenerative illnesses.

## Conclusion

The concentration of 2-aminoethyl dihydrogen phosphate normalized by the concentration of taurine in blood plasma reliably distinguished blood samples of early-stage AD patients from controls in a blinded analysis. If verified with larger sample sizes, the quantification of 2-aminoethyl dihydrogen phosphate could potentially assist in the diagnosis of early-stage Alzheimer’s disease when used in conjunction with the patient’s CDR score and other potential AD biomarkers.

## Abbreviations

Aβ42: amyloid-β-42
AD: Alzheimer’s disease
CDR: Clinical Dementia Rating
CE: collision energy
CSF: cerebrospinal fluid
FDA: U.S. Food & Drug Administration
GPI: glycosylphosphatidylinositol-anchored proteins
IUPAC: International Union of Poor and Applied Chemistry
NFL: neurofilament light protein
NFTs: neurofibrillary tangles
P-tau: phosphorylated-tau
PD: Parkinson’s disease
T-tau: total-tau
TCA: trichloroacetic acid
TNSALP: tissue-nonspecific alkaline phosphatase
